# Computational Commensality: From Theories to Computational Models for Social Food Preparation and Consumption in HCI

**DOI:** 10.3389/frobt.2019.00119

**Published:** 2019-12-05

**Authors:** Radoslaw Niewiadomski, Eleonora Ceccaldi, Gijs Huisman, Gualtiero Volpe, Maurizio Mancini

**Affiliations:** ^1^CONTACT Unit, Istituto Italiano di Tecnologia, Genoa, Italy; ^2^InfoMus Lab, DIBRIS, University of Genoa, Genoa, Italy; ^3^Digital Society School, Amsterdam University of Applied Sciences, Amsterdam, Netherlands; ^4^School of Computer Science and Information Technology, University College Cork, Cork, Ireland

**Keywords:** commensality, food, food recognition, HCI, social signal processing, embodied interfaces, social robots, augmented experience

## Abstract

Food and eating are inherently social activities taking place, for example, around the dining table at home, in restaurants, or in public spaces. Enjoying eating with others, often referred to as “commensality,” positively affects mealtime in terms of, among other factors, food intake, food choice, and food satisfaction. In this paper we discuss the concept of “Computational Commensality,” that is, technology which computationally addresses various social aspects of food and eating. In the past few years, Human-Computer Interaction started to address how interactive technologies can improve mealtimes. However, the main focus has been made so far on improving the individual's experience, rather than considering the inherently social nature of food consumption. In this survey, we first present research from the field of social psychology on the social relevance of Food- and Eating-related Activities (F&EA). Then, we review existing computational models and technologies that can contribute, in the near future, to achieving Computational Commensality. We also discuss the related research challenges and indicate future applications of such new technology that can potentially improve F&EA from the commensality perspective.

## 1. Introduction

Food and drink consumption is a vital human activity aimed at providing the body with nutrients that are necessary for survival. What is more, eating and drinking are also highly social activities that take place, for example, around the dining table at home, in restaurants, or in public spaces. People use food to regulate their own and others' emotions, for example, by offering food to cheer others up or by eating some particular food they associate with positive memories. Humans learn that food can have a social and emotional meaning from a very young age, for example, by associating food offering with soothing (Hamburg et al., 2014). Food-related interaction, often referred to as “commensality,” is very important for personal health and well-being (e.g., Grevet et al., 2012).

Given the importance of food consumption, researchers in human-computer interaction (HCI) and artificial intelligence (AI) have recently started to address how interactive technologies can improve mealtimes. For example, devices like sensor networks or connected appliances offering multi-sensory eating experiences (Kortum, 2008) are increasingly entering the processes of food preparation and consumption, while virtual agents (Gardiner et al., 2017) and robot companions (Baroni et al., 2014) are used to motivate children to eat more healthily. The variety of the topics related to *Food- and Eating-related Activities* (F&EA) has attracted researchers' interest from several AI-related disciplines: from computer vision to multimodal interaction and from positive to social computing, as demonstrated by the recently born series of workshops titled “Multi-sensory Approaches to Human-Food Interaction” and the “ACM Future of Computing & Food Manifesto^1^”.

**Figure 1:**
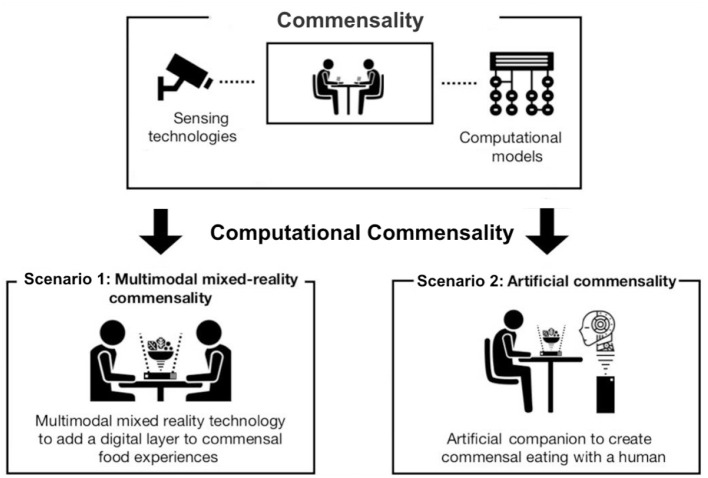
From commensality to computational commensality. We introduce research on “traditional” commensality in section 2, the papers related to the CC Scenario 1 in sections 4 and 6, and to the CC Scenario 2—in sections 3, 5, and 6.

However, research in AI and HCI and technologies dedicated to F&EA often focus on food (or eating) itself (e.g., food recognition and sensory augmentation) rather than on its social dimension. In this work, we introduce the concept of *Computational Commensality (CC)*^2^ to gather different attempts to computationally address various social aspects of food and eating. CC extends commensality in humans (see Figure 1), which is *the practice of sharing food and eating together in a social group* (Ochs and Shohet, 2006) by introducing technology as a “social glue” for food-related interaction. CC will focus on creation of rich physical or mediated multimodal interaction between two or more agents (being humans, or humans and machines) which may enable or enhance outcomes of the “traditional” commensality (i.e., in the sense of Ochs and Shohet's definition) studied so far mainly by psychologists and sociologists. CC needs, for example, F&EA recognition modules as building blocks to create food-related interaction. However, it goes beyond these topics already extensively studied in HCI and AI. It must also be distinguished from the other food-related concepts recently proposed, such as *gastroludology* (Chisik et al., 2018) and *human–food interaction* (Comber et al., 2014; Altarriba Bertran et al., 2018). The first one focuses on experiences involving playing with food (e.g., games). Indeed, such experiences can sometimes be social, e.g., when two or more persons use the technology to feed each other (Mehta et al., 2018) (see section 6.1 for other examples), but still the main focus is on sensorial, playful experience with food and the technological innovation enabling it. In this sense, Mueller et al. (2018) proposes considering the eating activity “as something not serious, with neither a clear goal nor real-world consequences.” The second one mainly investigates the individual experience, and rarely considers social context (although we present some recent interesting works in this field in section 5).

In our view, CC may appear in two main scenarios. First of all, two (or more) humans can use technology to enable or enhance human-human interactions during meals. Examples can be: using technology to enhance co-located social dining (e.g., Ganesh et al., 2014), or using tele-dining technology to enable social interaction between people who do not share the same physical space (e.g., Nawahdah and Inoue, 2013). In the second CC scenario, human(s) interact with an artificial companion, such as a social robot (e.g., Khot et al., 2019) during meal time. The companion uses sensors and computational models of commensality to guide its behavior toward the human interlocutor. In both cases, computational models can also be used to analyze and quantify the interaction during meals, for example, by detecting quantity of food consumed together or identifying the social roles at the table.

The main goal of this article is 2-fold: (1) we discuss psychological and sociological studies on the social aspects of food and eating activities, showing how they can be exploited to create CC; and (2) we present computational models, devices, and applications focusing on their social dimension, illustrating how they could be used in CC scenarios.

In the next section we will review contributions from social psychology dealing with food related interaction and social influence on food behavior, and we will illustrate recent HCI and AI works that deal with food preparation and consumption. In section 3, we will start our survey by illustrating existing works on food and eating recognition, which is probably the food-related topic most explored in computer science, with applications ranging from food production to virtual dining experiences. Existing solutions are usually based on computer vision and machine learning techniques, although other modalities such as audio have been sometimes explored. The most recent trends include the application of deep learning techniques for life-logging. We will present works dealing with human movement tracking and monitoring in food-related activities—for example, the recognition of drinking and swallowing actions from multimodal data coming from wearable sensors, audio or visual devices. In section 4, we will turn our attention to systems applying these technologies to provide physical or psychological support in eating activities. For example, systems offering physical assistance (e.g., for physically impaired people), mainly using robots, cooking assistants (e.g., in augmented reality), or serious games aiming to change bad eating habits. In section 5, we will discuss systems that use similar techniques with the aims to manipulate, augment, or enhance eating and drinking experiences through multimodal technologies. For example, several devices have been designed and tested to detect and simulate odors to be presented during food consumption as an additional sensory cue, while in other cases dining tables enhanced with projection mapping visualizations have been developed. These efforts provide insights on how technology can be introduced in dining activities to enhance the sensory experience of food and drink. In section 6 we will outline systems that use technology to, in a broad sense, enable, or stimulate interaction during food preparation and consumption. This includes multi-user games, robot-mediated physical interaction, as well as tele-dining systems. Most of these works already contain some aspects of CC, as they provide the technology and computational models to enhance or extend the human-human interaction around the food.

**Figure 2:**
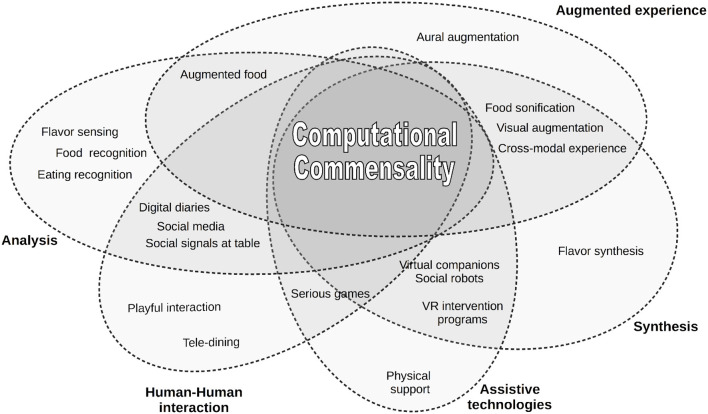
Landscape of topics discussed in this survey which are relevant to Computational Commensality.

Despite the long list of topics related to eating and AI we present in our survey (see also Figure 2), we believe that the investigation of the link between social aspects of F&EA and technology has just started. The variety of possible applications in this field is enormous, pushing this discipline to grow up quickly in the near future. We will conclude the paper by discussing some possible future research directions in section 7.

### 1.1. Selection of Sources

Given the novelty of the topic and its inherently multidisciplinary nature, we drew on many different sources from different disciplines and kept an open perspective in the selection of literature relevant to the current survey. Nevertheless, our main focus was on computational technologies that either had a direct relation with social dining practices (e.g., tele-presence dining), or computational technologies that would be part of or would enable such practices (e.g., computer vision, food recognition). For this reason, in preparing this survey, we mainly focused on “technology-oriented” online libraries, such as the IEEE Xplore Digital Library and the ACM Digital Library. Our initial search focused on work published in the past 5 years, to come up with the works we surveyed in sections 3–6. The following search terms were used: *commensality, eating, dining*, and *food* (see Table 1 for details). Our initial search resulted in an initial selection of 2174 (i.e., the total number of ACM and IEEE references from 2014 to 2019, see Table 1). To get a more complete overview of the field, in the next step, we searched for relevant sources cited in the papers from the initial selection. This process resulted in an additional selection of 3040 sources (Table 1). It is important to notice that not all papers in this pool deal with food-related technology. For instance, several papers contain the keyword “eating” in used as a verb in the title, e.g., “how to have the cake and eat it too,” without addressing eating-related research questions.

**Table 1:** The total number of the papers per keyword found in the online libraries (in parenthesis the number of the papers cited in this survey corresponding to given period and the source).

**Keyword**	**ACM digital library**		**IEEE Xplore digital library**	
	**2014–2019**	**2010–2019**	**2014–2019**	**2010-2019**
Commensality	13 (5)	17 (6)	1 (0)	1 (0)
Eating	322 (14)	466 (19)	670 (6)	926 (10)
Food	971 (10)	1299 (17)	—	—
Dining	66 (0)	106 (3)	131 (1)	225 (3)

*The research was performed on 23rd of August, 2019*.

In addition to this, we also relied on sources from psychology and social sciences (i.e., Frontiers in Psychology and Psychological bulletin) and from Appetite. In doing so, we leveraged the aforementioned keywords, often combined with: *social facilitation, social comparison*, and *social context*. The final number of sources used in this survey is indicated in parentheses in Table 1. It is important to stress that our intention, when preparing this survey, was rather to show the variety of topics relevant to CC than performing a systematic survey of one research field. For example, we do not aim to enumerate all papers on serious games for changing eating habits published in the last 5 years, but we show a broad spectrum of works we deem relevant to CC systems and applications. Consequently, this survey is different from previous attempts of reviewing the existing works (see next section 1.2 for more detail), which usually focus on one aspect of food-related technology only.

### 1.2. Related Work

Min et al. (2019) proposed a survey on Food Computing, defined as an interdisciplinary field addressing food-related studies via computer science. In their view, Food Computing applies computational approaches for acquiring and analyzing heterogeneous food data from various sources for perception, recognition, retrieval, recommendation and monitoring of food to address food related issues in health, biology, gastronomy, and agronomy. Furthermore, Food Computing is conceptualized as a collection of new methodologies and technologies for food science. According to the authors, Food Computing involves several steps. It requires data collection coming from different sources (e.g., social media, leveraging pictures and videos posted by users) and analysis carried out, for example, through machine learning or data mining techniques. At the same time, Food Computing has several applications, from food perception to recognition and from food recommendation to intake monitoring. The authors conclude by illustrating future directions and challenges of Food Computing. Although they mention that Food Computing might be involved in human behavior understanding especially in terms of the interaction of humans with food, Food Computing does not explicitly deal with the social dimension of food.

Shifting to Human-Computer Interaction (HCI), Grimes and Harper (2008) examined the literature on food and technology, pointing out that most of the existing works fall into two main categories: (1) technologies to solve food-related issues (for instance by helping inexperienced cooks to prepare a dish), and (2) technologies to modify the user's bad eating habits, namely “corrective technologies.” In their view, HCI should also focus on the pleasurable and socio-cultural aspects of eating, and they introduce the concept of *celebratory technologies* “that celebrate the way that people interact with foods” (Grimes and Harper, 2008). Within this aim, according to the authors, several concepts related to eating should be explored, such as creativity, pleasure, nostalgia, gifting, family connectedness, trend-seeking, and relaxation. In the last part of their work, they describe challenges and provide a possible research direction toward this new type of technology. Interestingly, they point out different “social” aspects of food, such as food *gifting*, which has a symbolic social function. Comber et al. (2014) illustrate how food practices have gained importance in HCI, building what is called Human Food Interaction (HFI). The main areas of interest in HFI are health and wellbeing, sustainability, food experiences, and alternative food cultures. In this, HFI differentiates from commensality as the commensal experience seems not to be the core of HFI. HFI is in fact more focused on food practices as socio-cultural artifacts, examining cultural environmental and political aspects of human-food interaction. Furthermore, a new body of research on the experience of food, known by the term of *gastrophysics* (Spence, 2017b), has grown, describing the many factors driving food perception and enjoyment. The *scientific study of dining* illustrates how all sensory modalities drive food experience, along with the context in which the meal is consumed. In this sense, the social dimension can influence the food experience as much as the edible elements on the plate.

## 2. Food as a Social Phenomenon

Food itself has an inherently social and emotional meaning. As such, it has been the subject of psychological and socio-psychological studies aiming at investigating it as a social phenomenon. Studies on traditional commensality investigated several interaction-related phenomena, such as conversational patterns in families (Laurier and Wiggins, 2011). Research on conversation analysis has drawn its attention to F&EA, for example, preparing (Paay et al., 2015) and sharing (Sterponi, 2003; Goodwin, 2007; Mondada, 2009) a meal. Research on workspace interaction has demonstrated how food and beverages, for istance, coffee, can have important communicative functions, with sipping becoming a cue to turn taking during conversations (Laurier, 2008). Furthermore, meal time is a key moment for addressing gestural interaction, as shown by Nansen et al. (2014). Other researchers focused on the cultural aspects of joint food practices (Fischler, 2011). They showed how the importance of the social dimension of food changes across cultures, being more relevant for French and Italian than for U.S. eaters. Ferdous et al. (2016b) illustrated how commensality and technology are often blended together, with technologies such as smartphones and tablets often being included in family dinners. The social dimension of eating is further explored by works on *social comparison*. In psychology, the term *social comparison* indicates how people spontaneously compare themselves to others as a means of self-evaluation (Festinger, 1954). Researchers have shown that mealtime is often a chance for social comparison (Polivy, 2017), with food perception and intake being influenced by others' presence (Polivy and Pliner, 2015). Social psychology has demonstrated how being with others can affect mealtime in terms of food intake (Bell and Pliner, 2003; Herman et al., 2003; Paquet et al., 2008; Hermans et al., 2009, 2012; Howland et al., 2012), food choice (Stroebele and De Castro, 2004; McFerran et al., 2009; Prinsen et al., 2013), calory consumption (Young et al., 2009), and taste perception (King et al., 2004; Poor et al., 2013) and how this influence is modulated by group membership (Cruwys et al., 2012), relationship status (Salvy et al., 2007), and, interestingly, that such influence still holds in virtual eating scenarios (Fox et al., 2009).

According to Simmel (1997), the meal has an *immeasurable sociological significance*. In fact, it is well known that, from a very young age, humans learn that eating is more than just introducing food into the body. Hamburg et al. (2014) illustrated how infants learn the soothing effect food can have and, more interestingly, according to the scope of this survey, that food offering can be a way to show empathy for others' distress. The term *comfort food* refers to those foods whose consumption provides consolation or a feeling of well-being (Spence, 2017a). In this view, food has the possibility of fostering positive emotions. Troisi et al. (2015) stated how food can have the capability of making people feel socially connected and call this property the *social utility* of food. Furthermore, they stress the idea of food as a social surrogate, demonstrating how (comfort) food choice is often associated with social isolation. As Connor and Armitage (2002) claimed, social psychologists contribute to food research by addressing topics as factors in food choice (McFerran et al., 2009), dietary change (Lange et al., 2018), weight control and well-being (Utter et al., 2018), snacking (Schüz et al., 2018), and food and self-presentation (Herman et al., 2003). In this context, the most thoroughly explored topic is what social psychology defines as the *social facilitation of eating* (e.g., Herman, 2015, 2017). This term describes the influence of presence of others on food intake. Diary studies have shown how meal size increases with the degree of intimacy with meal companions (De Castro, 1994) and how the presence of others models food intake, by acting as a guide on what and how to eat (Cruwys et al., 2015).

### 2.1. Summary

In our view, CC is a multidimensional phenomenon. So, the social and psychological aspects of F&EA should be taken into consideration when proposing computational models aimed at augmenting, analyzing or simply recognizing commensality. On one hand, several results mentioned in this section can relatively easily be replicated to check whether the technology-enhanced interaction can foster similar (positive) outcomes on interactants as it was observed in “traditional” commensality. This can include, for example, experiments on the quantity of food intake, or the degree of intimacy experienced by interactants. Work on socio-affective values of F&EA might contribute to new computational techniques for analyzing social interaction by relying on social, commensality-related cues. For instance, as humans can infer relationship statuses from observing people sharing food (Erwin et al., 2002), we could envisage technologies able to exploit F&EA-related cues in a similar way.

On the other hand, some existing computational models can be useful to quantify the social interaction around the table, and, thus, to address important research questions in the field. An example of how CC could benefit, e.g., social psychology, could consist in using a quantitative approach to detect the person at the table who is marginally participating in the conversation while eating (e.g., from their gaze behavior and amount of food intake (see Otsuka and Inoue, 2012).

## 3. Technology for Food and Eating Recognition

In order to account for the role of food and food related behaviors as non-verbal social signals, technologies must be able to afford their automated recognition. Technologies able to detect food related activities (for instance swallowing or drinking) contribute to CC because interaction in ecological settings often revolves around food (e.g., parties, dates, meetings). In particular, in the CC scenario 2, social robots and virtual agents would benefit from being able to detect when human interactants are involved in a F&EA, such as taking a bite of food or sip of a drink. Such a F&EA during interaction could influence, for example, turn-taking, which should be taken into account by the social robot or virtual agent when it serves as a companion during food consumption. In addition, the artificial companion might be able to detect the type of food the human interactants are consuming and can comment on the qualities of the food in conversation.

This section reviews existing studies on food and eating detection: going from computer vision algorithms for food recognition (both on food image datasets and on pictures or video captures in the wild) to automatic trackers of eating activities (e.g., automatically detecting food intake quantity and speed).

### 3.1. Food Detection and Recognition

Several algorithms and techniques for food detection and recognition rely on the huge amount of pictures people share on social media every day. It must be noted that such pictures are mostly egocentric pictures, meaning that they are taken from the point of view of the user. They can be automatically captured by wearable cameras (e.g., for medical purposes) or taken by users of mobile devices (e.g., smartphones). They usually have low quality and poor framing, so the objects to be detected (e.g., plates) are far from the center of the picture, have scarce illumination, are deformed by the camera lens, and so on. These pictures are the most commonly shared by users on social media or on instant messaging apps, making them particularly interesting for automated analysis. To deal with food pictures, authors can exploit several existing datasets on food: *ILSVRC 2013* by Russakovsky et al. (2015), *Food101* by Bossard et al. (2014), *UECFood256* by Kawano and Yanai (2014), or *Egocentric Food*^3^. The resulting recognition models showed high accuracy in locating food, both in traditional and egocentric pictures, when there is no overlap between objects. Such approaches and resources, although not directly aimed at investigating commensality, are required steps toward analyzing human behavior during mealtime.

Bolaños et al. (2013) implemented a technique, based on machine learning, for the labeling of huge amounts of images. The algorithm they presented, based on a Hierarchical Sampling (HS) method, determines whether or not a plate is present in an image. According to the test on about 90k images, the algorithm can label all images in about 40 min in a totally unsupervised setting. Similarly, Ciocca et al. (2017) created the UNIMIB2016 dataset consisting of 3616 food instances belonging to 73 food classes (e.g., “pasta,” “pizza,” “yogurt”). The dataset is manually annotated to separate the food from the background. The authors also performed the automated recognition of food types using K-Nearest Neighbors (k-NN) and Support Vector Machine (SVM).

Aguilar et al. (2017) aims to build an application for automatic food habits tracking. It is a multi-labeling task, that is, a machine learning problem in which there are multiple output labels (instead of a single one), and it is solved using a Convolutional Neural Network (CNN). Results have shown good recognition rates also for recognizing ingredients of recipes that were not present in the training set.

Herranz et al. (2018) propose to take into account context and external knowledge in automated food detection. Context is, for example, the location, date, and time a food picture was taken. External knowledge includes food recipes, nutrition information, restaurant information, and food images and videos. In this framework, the authors review existing works on multimodal cuisine analysis, focusing on food recognition in restaurants. As mentioned before, studies exploiting egocentric pictures often have to deal with poor image quality; despite this, egocentric pictures (due to their great availability) are still leveraged, as in Jia et al. (2018). This work illustrates the development of the *eButton*, a small box containing a camera and a motion sensor. Using it, the authors showed that, even if the quality of egocentric images is lower than that of a smartphone, still it allows for food detection. To do that, they chose to exploit an existing CNN, the Clarifai CNN (Zeiler and Fergus, 2014), and compared food recognition on images captured by the eButton vs. images belonging to the Food-5K dataset (Singla et al., 2016). Results showed that the performances of the CNNs are comparable.

It is worth noticing that, as a consequence of the works we have described above, there already exist solutions for food recognition that take into consideration the context (e.g., location, like in Herranz et al., 2018). However, it seems that the social context is not considered (yet). The information about the group (e.g., the number of people involved) and the group bonds (e.g., their level of intimacy) can be contextual information helpful to recognizing some type of food. For example, some types of food are eaten usually in close company, such as, birthday cakes, raclette, fondue, or Korean BBQ, while the others are more often eaten alone, for istance, fast food. This example demonstrates the need to introduce models of CC in food recognition.

### 3.2. Eating Activity Detection and Recognition

As far as eating activity recognition is concerned it must be noted that activities linked to food preparation present a high intra-class variability, as highlighted in Stein and McKenna (2013b). They observed that recognition would be possible if large data-sets were available, but this is not the case with food preparation activities. For this reason, they present work in which activity recognition is carried out by performing a training on a limited amount of data, collected in the publicly available *50 Salads dataset* presented in Stein and McKenna (2013a) and Chen et al. (2017). They compared two approaches: classifying (e.g., SVM, k-NN) activity of single users and then combining the results vs. performing a combined classification. They argued that the first one gives the most accurate results as it takes into account intra-user variability. Features were extracted from accelerometers attached to objects and from environmental video data (e.g., a camera framing the cooking area from top).

Wearable sensors can be used for eating recognition and detection, but they are typically intrusive. For instance, Bi et al. (2014) exploited a necklace-like device and a smartphone to capture throat sounds, and applied machine learning techniques (e.g., kNN, SVM) to determine the eating-related user activity (e.g., chewing, swallowing, breathing). The device could be applied to monitoring what and how people eat during the day to better address food-related health problems like dysphagia and indigestion. Their system was based on a microphone and on the extraction of acoustic features to be later used for training and classification of eating-related activities, which reached over 95% accuracy. Amft and Tröster (2008), similarly to Bi et al. (2014), developed an on-body sensing approach to detect three key activities during food intake: arm movements, chewing, and swallowing. They applied Hidden Markov Models (HMM) on inertial sensors data to recognize arm movements. Chewing was recognized by analyzing the produced sounds. Swallowing was detected from the fusion data captured by two sensors: a surface EMG sensor and a stethoscope microphone. Moreover, Mendi et al. (2013) propose an application for eating activity recognition based on an accelerometer placed on the user's wrist, providing the user with information on the total number of bites, bites-taken rate and eating speed. The application is based on acceleration peaks detection and sends real-time warnings to the user when the eating speed is over a given threshold.

Rahman et al. (2015) highlight that eating is difficult to be accurately and unobtrusively recognized and analyzed. Worn sensors, for example, are deemed as uncomfortable and, in their opinion, they should be avoided. As an alternative, they propose to use Google Glass to track head movements, and they demonstrate that the captured inertial data (i.e., accelerometer, gyroscope, and magnetometer) from this device are informative enough to automatically recognize users' eating activity with traditional machine learning techniques, such as k-NN and RF. Interestingly, similarly to Stein and McKenna (2013b), Rahman et al. (2015) also see as the primary application of their work the possibility to better monitor and cure chronic diseases like obesity and diabetes. Other approaches to overcoming the intrusiveness of wearable sensors for eating recognition have been proposed. An interesting approach was illustrated by Chang et al. (2006), who designed a “diet-aware” dining table that used weight sensors and radio frequency identification (RFID) readers in order to measure food intake of diners at the table. The combination of weight sensors and RFID tags embedded in food containers enables the detection of food being moved from a central container to an individual's plate, allowing measures to be taken during a multi-party dinner. A first small-scale evaluation of the system showed recognition accuracy of food transfer from a central container to an individual plate and eating events to be around 80%. Another example of a device for the monitoring of food intake in ecological settings was proposed by Fontana et al. (2014), who developed a wearable system composed of a jaw motion sensor, a hand gesture sensor, and an accelerometer. The system is integrated with a smartphone equipped with a food intake recognition module which uses dedicated sensor fusion and pattern recognition techniques. The device was validated in real-life conditions over a one-day period by 12 subjects. Results showed that the system was able to detect food intake with an average accuracy of 89.8%.

An interesting contribution to CC was proposed by Kiriu et al. (2017) who, using the data from a smartwatch and a smartphone, are able to recognize whether a person is eating alone or in company, with an accuracy of 96%. The data consist of kinematics data (e.g., from 3D accelerometer) and several metrics of the smartphone. At the moment, this approach was tested only on a small dataset of 20 participants, but it showed a very interesting direction of research to be more deeply investigated in the framework of CC.

With the aim of addressing eating activities ecologically, while preserving their social dimension, it might be necessary to discriminate eating from speech. The goal of the work illustrated by Hantke et al. (2016) and Hantke et al. (2018), which was part of the EU iHEARu Project^4^, was in fact to demonstrate that Automatic Speech Recognition can be improved by introducing the automatic recognition of eating conditions. To do that, they collected the iHEARu-EAT audio/video database, featuring 1.6k utterances of 30 subjects, 6 food types, and read/spontaneous speech. The authors performed a number of experiments in different conditions to discriminate between normal speech and eating speech, and to detect the type of food that was eaten while speaking. Results were positive, though the authors highlighted that the accuracy of detection, based on SVM, was strictly linked to the training that was carried out on some specific food (apple, nectarine, banana, Haribo Smurfs, biscuit, and crisps).

### 3.3. Future Developments Toward Computational Commensality

Overall, the existing technologies for food and eating recognition are not yet ready to be exploited in real-life applications. In a recent study, Alharbi et al. (2017) addressed the challenges of wearing devices (video camera, neck-worn sensor, and a wrist-worn sensor) for food activity monitoring to support weight management, mainly in terms of comfort of wearing a camera, and privacy. Results showed that participants had many concerns about privacy and had the feeling of a *social stigma* of wearing electronic devices that could worry other people around them.

Eating recognition might benefit from introducing the social context to the recognition models. One can imagine the recognition systems, in which subject data coming, for example, from an accelerometer placed on their wrist (e.g., similar to Mendi et al., 2013), is compensated by the data coming from similar devices placed on wrists of their eating companions. Research has in fact demonstrated that people eating together tend to mimic their companions' food intake, for instance in terms of bite rhythm (Hermans et al., 2012). Indeed, in the commensal scenarios, models for the recognition of eating activities should take into account social dynamics between the interaction partners, for istance, conversation turn-taking, social relations between the eaters (e.g., leadership, level of intimacy) but also other contextual data, such as the place of eating (e.g., fast food or an exclusive restaurant).

## 4. Assistive Technology

Much attention has been oriented toward the development of technologies to provide support in eating activities or during food preparation (as in Mennicken et al., 2010; Angara et al., 2017). Such technologies include systems offering physical support and assistance (e.g., for physically impaired people), mainly through the use of robots. A separate category consists of mobile apps that monitor and help to change the eating habits and increase overall well-being. Here we can distinguish two subcategories: some of the systems can supplement therapy related to some concrete health problem such as diabetes, while others can be used to improve the general habits, for example, by promoting a balanced diet, and, consequently, increasing the well-being of users. With a similar goal, several virtual and robotic assistants and serious games were developed. In particular, the gamification approach is a very popular method used specially in systems dedicated to young end users. All these applications focusing, at least at the moment, mainly on health and well-being related goals, are relevant to CC as they may relatively easily become CC use cases, in which one or more humans enter into interaction with a socially intelligent system (tutor, coach, assistant). One can, for example, easily imagine a virtual character which would not only assist the user by explicitly instructing her about healthy eating, but create a rich and fruitful social interaction, which can, indirectly, influence the well-being of the user and consequently her eating habits, too. To reach this aim, overall social skills of machines need to be improved.

### 4.1. Artificial Companions

Several systems have been designed to assist humans in changing their eating habits by leveraging the communicative (and sometimes affective) skills of humanoid assistants, be they virtual (e.g., embodied agents) or physical (e.g., robots). They usually address some very specific populations, such as hospital patients, children, or the elderly, as those groups often benefit from healthier life style, including healthier eating habits. Angara et al. (2017), proposed an interactive kitchen assistant giving health recommendations. Interestingly, the virtual agent's food-related interaction with the user was enriched by taking into account the user's food habits and cultural food preferences. Gardiner et al. (2017) evaluated the use of such technology to promote healthier eating behaviors. A virtual assistant is able to interact face-to-face with a user providing personalized dietary suggestions and health information (e.g., food recipes) and asking food-related questions to the user. A study on 61 female participants using the system during a 30-day span demonstrated a decrease of negative eating habits (e.g., drinking alcohol) and an increase of positives ones (e.g., fruit consumption). Such assistants do not need to have a human-like appearance, as in the case of the work by Pollak et al. (2010), who developed *Time to Eat*, a mobile virtual pet game designed to enhance healthy eating habits in teenagers. The pet sends healthy eating daily reminders to the user. In response, children take photos of their meals and snacks, of which the “healthiness” is evaluated by the app, which in turn influences the pet's emotional state (e.g., junk food corresponds to sadness, healthy food corresponds to happiness). Again, an evaluation involving 53 children showed that the app had positive effects on their eating habits. Parra et al. (2018) proposed an interesting combination between a human-like virtual assistant and crowd-sourcing. They developed an app with an e-assistant able to discuss the preparation of a meal with a human user. Then, the user can upload a photo of the meal and receive an evaluation provided by another user of the same app. The final system was evaluated on 59 patients, who found it useful, easy to use, and helpful in maintaining tasks “related to their diet.”

With the aim of solving issues caused by solitary eating, Takahashi et al. (2017) proposed a virtual co-eating system allowing enjoyable conversations related to the meal, as well as typical daily conversation to be maintained. A virtual character is displayed on a mesh fabric, and the character has an embedded facial expression recognition module. The results of a preliminary evaluation of the system are particularly interesting in the CC context, as 4 out of 5 participants reported improvement when comparing the eating alone condition with the one in which they ate together with the virtual character.

Assistants promoting healthier eating styles can have physical bodies, as in the case of robots. Baroni et al. (2014) evaluated the effect of a humanoid robot on children's dietary choices. The robot can successfully persuade children to eat more fruit and vegetables by communicating verbally (modulating the voice, and using encouragements) and non-verbally (through gestures, proxemics, gaze). Eating habits in young children are also addressed by Randall et al. (2018) with their Health-e-Eater, a sensor-equipped plate and a simple robotic companion which motivates and educates children during meals. Health-e-Eater is a low-cost robot architecture based on a Raspberry Pi 3, equipped with LEDs, a vibration motor, a servomotor, and a speaker. LED lights and verbal messages are supposed to focus the attention of the child on the food, encouraging and rewarding them when a healthy eating style is detected. McColl and Nejat (2013) proposed an assistive robot designed to cognitively stimulate and engage the elderly during eating. Starting from existing studies on the role of the interaction during meals in the improvement of dietary intake (e.g., Schell and Kayser-Jones, 1999), they designed an autonomous robot able to detect the amount of food intake while interacting with the user, both verbally and non-verbally. Some of the robot utterances are directly related to the eating itself (e.g., encouragements), while the others are aimed at enhancing the interaction (e.g., greetings, telling jokes, laughing). An exploratory study was conducted on a group of elderly, and results showed that participants felt engaged, enjoyed themselves, and cooperated with the robot in response to its prompting behaviors.

**Figure 3:**
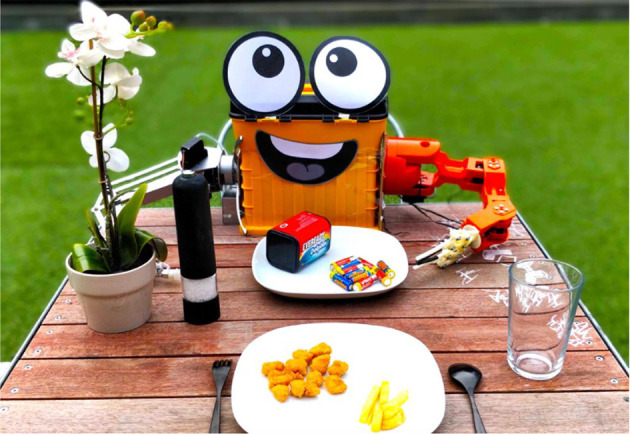
FoBo—a robotic dining companion. Reproduced from Khot et al. (2019) with permission from the authors who hold the copyrights.

In general, we believe that social robots are particularly appropriate to become commensal partners, but very little research has been presented on this topic so far. Within the aim to exploit the positive outcomes of commensality, Khot et al. (2019) recently proposed a robotic dining companion called FoBo (see Figure 3). The role of this robot is to create playful and entertaining interactions around a meal with no clear “real-world” goal. So, it does not instruct or correct human's behavior but, instead, for istance, it “consumes” batteries, performs sounds related to eating (e.g., burping and purring), as well as mimics some human behaviors.

### 4.2. Virtual and Augmented Reality

Virtual and augmented reality are also used to create situations aimed to change the human eating habits. For instance, Celikcan et al. (2018) proposed the Virtual Cafeteria–VR immersive environment designed for nutrition education of adolescents. The virtual buffet offers a large selection of foods and drinks covering the three meals. The users create their own meals and can pick any portion for any available food. At the same time, they are given age appropriate recommendations on healthy eating and recommended portion sizes of each food group. The data related to the user activity are collected, so as soon as the session ends, the detailed nutritional information of the assembled meal is immediately available. In a similar vein, the Virtual Food Court (Nordbo et al., 2015) is a VR environment for studying humans' food choices in the context of policy-based interventions. It was successfully used to analyze the effects of introducing taxes for unhealthy food on food choices. Narumi et al. (2012) propose a system based on Augmented Reality (AR) and a Head Mounted Display (HMD) to influence food consumption and the perception of satiety by exploring the phenomenon of cross-modality. The system allows for the augmentation of food volume using the shape deformation techniques to give the user the impression of consuming more than she does in reality. The evaluation shows a significant effect of size (enlarged vs. shrunken) on the quality of food consumed. Participants ate significantly more food when the size was virtually decreased as compared to the condition of virtually increased food. According to the authors, such a system might be useful in treating obesity. Additional evidence for positive effects of VR on helping people in acquiring more healthy eating habits is provided by Tuanquin et al. (2018). The authors used VR to change the visual and olfactory appearance of food items in order to gradually change a person's eating preferences toward more healthy food choices. The goal was to help individuals with eating disorders through VR cue-exposure therapy (CET). Results of a first study, in which participants were presented with actual and virtual chocolate chip cookies, showed that the VR setup was able to successfully induce food craving and the urge to eat the cookie. According to the authors, the VR setup shows potential to aid in CET by presenting virtual food cues during therapy. Indeed, VR is increasingly recognized as a potentially useful tool to study human behavior regarding food choice (Nordbo et al., 2015; Ung et al., 2018), and food cravings (Ledoux et al., 2013), as well as research into the sensory aspects of food selection and consumption in general (Stelick and Dando, 2018).

### 4.3. Dedicated Sensing Devices

As for other devices that can positively intervene in human eating habits, Hermsen et al. (2016) carried out an experiment involving a smart fork (i.e., a fork-shaped device augmented with sensors and actuators). The fork can provide real-time haptic and visual feedback to the user (Kadomura et al., 2013), for example, producing alerts if the user eats too quickly. Eleven participants who perceive themselves as “fast eaters” were asked to use the fork during 3 days. Most of them reported an increased awareness of their eating rate, and decrease of the eating speed. Drink-O-Mender by Ritschel et al. (2018) is able to sense the type and amount of drinks consumed by an adult, providing verbal advice depending on calories and nutritional values. For example, it may try to attract the person's attention toward the drinks with the lowest amount of calories.

### 4.4. Future Developments for Creation of Computational Commensality With Artificial Companions

Assistive technologies, at least at the moment, rarely explore the social bonds with their users. An interesting exception we mentioned above are the works by McColl and Nejat (2013) and Khot et al. (2019), where the robot builds an interaction for which the aim is not only functional (i.e., assistive) but also social. Future solutions may include, for example, tools for reciprocal assistance. Moreover, even if social aspects of eating are considered, it is usually limited to dyadic interactions. A robot bartender by Foster et al. (2012) is a rare example of an artificial companion able to deal with multiple humans in a dynamic social setting. Their robot is able to engage in multiple socially appropriate interactions at the same time when performing a task-oriented activity (i.e., serving drinks).

Similarly, immersive VR/AR systems currently focus on the individual experience. Systems such as the Virtual Cafeteria mentioned in this section can easily become multi-user social systems, where different users can interact and exchange their experiences regarding the food, similarly to how they do now using dedicated forums (see, e.g., Parra et al., 2018).

At the same time, it is important to stress that examples of artificial (eating) companions that do not have either an assistive or coaching role, are even more rare. Liu and Inoue (2014) propose a virtual eating companion that aims to be an active listener in order to support the generation of new ideas. According to the authors, the person who has a meal is likely to become an attentive listener, while the other interactant more likely becomes a speaker. Based on this assumption, the authors created a virtual character whose eating behavior is modeled on the quantitative analysis of actual dining behavior. For this purpose, recordings of multiple students eating together were used. Performing such analysis of human-human interaction is a good first step to create CC applications. Unfortunately, such works are still rare, especially when we consider papers that use technology to automatically quantify social interactions during meals.

## 5. Technology for Augmented Flavor Experiences

In the literature dealing with food and technology there is a large body of work on the use of technology to alter flavor experiences (Spence and Piqueras-Fiszman, 2013; Bruijnes et al., 2016). These works are grounded in research into the multi-sensory nature of flavor experiences (Velasco et al., 2018). The central notion is that flavor is a multi-sensory construct of which the percept results from a combination of information from several sensory channels (Auvray and Spence, 2008). A change to one sensory channel (e.g., the color of the food) can potentially influence the flavor experience of the food consumed. Several techniques exist that can be used to digitally alter the flavor experience of food. This is of interest to CC because such alterations could be used to create new social dining experiences, new ways of socially sharing food experiences, and can give robotic or virtual dining companions some form of control over actual food being consumed by human co-diners.

### 5.1. Visual Flavor Augmentation

Considering flavor as a multi-sensory construct, changing the visual appearance of food has been demonstrated to have an impact on flavor experiences (Zampini et al., 2007). A potentially interesting method to digitally alter the visual appearance of actual food is the use of projection mapping (Kita and Rekimoto, 2013). In one study, projection mapping was used to alter the visual appearance of yogurt. Colors, shapes, and animations were found to have the potential to change flavor experiences (Huisman et al., 2016).

### 5.2. Auditory Flavor Augmentation

**Figure 4:**
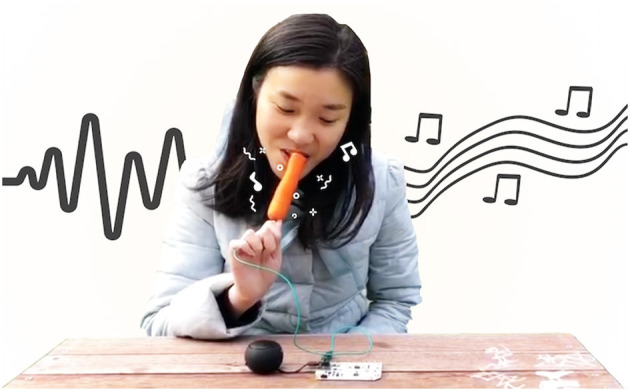
The user is playing with iScream! Reproduced from Wang et al. (2019) with permission from the authors who hold the copyrights.

Auditory feedback can be used to change the perceived texture of food, altering the overall experience of eating, say, crisps (Zampini and Spence, 2004; Koizumi et al., 2011). Wang et al. (2018) propose a five-keys framework for augmentation of the eating experience with sounds. According to them, (1) new sounds can be generated (that are different from the natural sounds of consumed food), (2) the natural sounds can be amplified, (3) removed, or (4) blended with other (food related) sounds. Finally, the sounds can also be (5) distorted. Within this framework, the authors propose the Singing Carrot, a platform for the exploration of food sonifications, which generates sounds when the user eats a carrot. The system detects food consumption through capacitive touch sensing, and the value of sensed capacitance is mapped to the frequency of a sinewave, resulting in eating sound that dynamically changes. A similar concept is used in the iScream! system (Wang et al., 2019, see Figure 4), which allows the use of a novel "gustosonic" experience of digital sounds which are automatically created as a result of eating an ice cream. It also uses capacitive sensing to detect eating actions, and based on these actions, it plays different sounds to create a playful eating experience.

### 5.3. Haptic Flavor Augmentation

Augmentation of haptic sensations, for example through electrical muscle stimulation, can create augmented experiences of food texture (Niijima and Ogawa, 2016). Iwata et al. (2004) in their research focused specifically on creating a simulation of mastication, using haptic technology. The biting force used to chew on real food items (e.g., a cracker) was recorded and used as data in the system to modulate the physical resistance provided by the haptic device in order to produce sensations of biting into different food items.

### 5.4. Chemical Flavor Augmentation

Ranasinghe et al. (2011) and Ranasinghe and Do (2017) highlight how taste and smell are the senses allowing us to remember emotions and feelings, as they directly influence our mood, stress, retention, and recall functions (Drewnowski, 1997). With this in mind, Ranasinghe and Do (2017) created the Digital Lollipop, a device that synthesizes taste (e.g., sweet, salty, sour, bitter, and umami) by applying a small electrical and thermal (i.e., applying heat vs. cool) stimulation to tongue. The Digital Lollipop consists of two silver electrodes, a sphere and a plate, and can generate square wave pulses with a current ranging from 20 to 200 μ*A* with frequencies in the range 50–1,200 Hz. The tongue must be placed between the electrodes. In an experiment presented in Ranasinghe and Do (2017) the authors observed that, by placing the electrodes on the tongue tip and sides, 90% of participants perceived sourness, 70% saltiness, 50% bitterness, and 5% sweetness (corresponding to the case in which the current was inverted). Some participants perceived a tingling, pineapple-like sensation when current increased. Tip and side stimulations exhibited slight variations in the observations, mainly related to the intensity needed to elicit the sensations, which was lower. Note, however, that there are specific challenges to using technology to address the chemical senses, such as the notion that people generally pay less attention to smells when they are engaged in another task as well as the need for using capsules to produce artificial scents (Spence et al., 2017).

### 5.5. Multimodal Flavor Augmentation

There are also multimodal approaches where digital augmentation is used to address multiple sensory channels at the same time. For example, Narumi et al. (2011) used a HMD to alter the visual appearance of cookies. An olfactory display was synchronized to the visuals to create the illusion of, for example, chocolate flavors. In another example, researchers used electrical stimulation of the taste buds on the tongue, in addition to lights, and an olfactory display to augment the flavor of an actual drink (Ranasinghe et al., 2017, see also Narumi et al., 2010; Ranasinghe et al., 2014, 2016).

One solution could be to create VR experiences that more carefully integrate with “real” experiences, by having actual objects (e.g., food or drinks) also be virtually represented in VR (Harley et al., 2018). Automatic computer vision-based food recognition solutions could be used in such an approach way to create compelling mixed reality experiences (Kanak et al., 2018).

### 5.6. Future Developments for Multi-Sensory Commensality

There is a large body of research on multi-sensory flavor experiences (for an overview see Auvray and Spence, 2008), and this research has since found its way into high-end restaurants such as Sublimotion^5^ and Ultra Violet^6^ (see Spence and Piqueras-Fiszman, 2014 for additional examples), where diners not only experience haute cuisine but also high-tech.

While the research discussed above does not directly bear on commensality, it can be argued that flavor augmentation and flavor synthesis have a potential future role to play in social communication and CC. For example, Ranasinghe et al. (2011) discuss how their flavor synthesizing technology could be used for flavor communication between remotely located individuals, creating a new kind of remote communication. Similarly, flavor augmentation technology could be used to share experiences around actual food items. Remotely located diners could potentially adapt the flavor experience of their companion's food to their own flavor experience (see also section 6.2). The potential future application of these technologies could be envisioned to be in the realm of social media communication and the sharing of experiences through social media, similar to how applications such as Instagram are now used to share visual aspects of food. Connecting such social food sharing to food printing technologies would create interesting forms of social “food messaging” (Wei et al., 2014).

Other applications of food augmenting technologies in commensal scenarios might include using food augmentation to communicate emotional and social states of interaction partners, for exmaple, by dynamically changing their food properties such as a color or by adding the relevant sonification. These alterations could be part of CC models that also drive social behaviors of robotic or virtual dining companions so that they can interact with humans through the food on the table.

## 6. Technology for Fostering Human-Human Interaction

Several technologies have been proposed that can be considered digital extensions of social activities. For example, job meetings can take place through video-conference (Jo et al., 2016) or artistic performances through Networked Music Performance (Rottondi et al., 2016). Similarly, technologies such as tele-dining platforms were proposed as a digital counterpart of eating activities. In this section, we describe technologies that are supposed to be able to deal with the social aspects of food and eating-related activities and which are designed to make eating more social as well as more enjoyable.

### 6.1. Serious Games and Playful Interaction

Particularly popular are solutions which combine educational purposes related to food intake with entertainment, often in the form of a serious game. Such games often introduce elements of competition or cooperation between two or more players, and thus, a social dimension to the activity. We4Fit by Pereira et al. (2014) is an example of such a mobile app that uses a gamification approach to modify the motivation of users to change eating habits and promote a healthy lifestyle. Interestingly, it is a rare example of a collaborative food-related game: in the game, the user (or a team) posts pictures of the consumed food. Other participants rank the photos indicating how healthy the photographed food is. At the end of each seven-day round, the sum of the ranks is used to establish the winning team. Playing in teams, according to the authors, should enhance the motivational effect, as the members of the team can influence each other to obtain a better final score. Another application that uses similar mechanisms of interaction between the users is called Foodie Moodie (ElSayed et al., 2018). This app promotes the awareness about the relation between the type of food consumed and the mood. It allows the users to keep track of what they eat and understand how it may affect their mood, as well as provides guidelines to other users about the possible interrelation between mood and food. A gamification techniques were also included in the app: first, the users can collaborate by adding and (re-)viewing the others' hints related to the topic. They may also compete with each other trying to obtain the highest total score on their tips. Other serious games use immersive virtual environments and AR. Ganesh et al. (2014) used interactive projection mapping to introduce game elements on children's plates during eating, with the goal of addressing children's reluctance to eat certain types of, predominantly healthy, food. The system is composed of two applications: one which changed the color of food items and one that awarded points and virtual badges for eating healthy items. The system was evaluated with children and their families at home. Observational data indicated that children ate food items they were otherwise reluctant to eat, and showed a playful attitude to food and the system. In addition, the system served to stimulate interactions between parents and children regarding healthy food intake. The authors considered the system's role in enhancing parent-child interaction and interactions between siblings to be particularly important. In this sense, it can be considered an example of commensal technology.

*You Better Eat to Survive!* by Arnold (2017) is a VR game in which eating real food becomes an input to control the narrative of the VR game. The players work in teams: when one player tries to realize some tasks in a virtual world, the other needs to feed him with real food to keep the first one “alive.” In the background, the system is able to detect the eating events via a microphone placed near to the first player's mouth. The VR game objective is to get rescued from an island. During its exploration, the player must keep himself alive by eating regularly. Otherwise, due to hunger, he loses consciousness, which is the end of the game. The team players can succeed in the game only if they collaborate, thus the game becomes a social experience. *Feed the Food Monsters!* by Arza et al. (2018) is a two-player AR game that uses chewing real food as an input to control the flow of the game. In order to achieve the goal (i.e., feed the virtual monsters that live in the stomach) the participants need to chew slowly. The participants can monitor each other's chewing behaviors through an interface that is displayed on their torso. Thus, they can also interact and guide each other to chew properly. AR is used to visualize the process of digestion though the means of using playful animations rather than showing the actual human anatomy.

The *Restaurant Game* (Orkin and Roy, 2010) is an example of a virtual commensality platform for human virtual agent interaction. The aim is 2-fold: it is designed to collect the data of human interactions, for example, when playing the a role of a customer or waiter in a 3D virtual environment, as well as to generate plausible behaviors of virtual agents. In the system, humans control characters from a first-person perspective using the mouse and chat. Agents are also able to interact and build a conversation both with humans and other agents. The behavior patterns of the agent are learned automatically from logs of the previous game sessions. Although it is not clear whether the previous game sessions include also the logs of human-human interactions in the VR system, such an extension would definitely be valuable in the context of CC.

Other examples of playful approaches to enhance interaction during dining using technology involve physical installations. One playful approach is presented by Mehta et al. (2018). The *Arm-a-dine* system involves two users both wearing a robot arm attached to their chests. A mobile phone camera is used to detect the facial expressions of the diners. For example, if a negative facial expression is detected the wearers own robotic arm will pick up a food item and present it to the wearer. If a positive facial expression, such as a smile, is detected the robotic arm of the partner will offer a food item to the person smiling. The central concept of the system revolves around taking away some bodily control in order to stimulate new kinds of social interactions around food consumption. One finding from a first exploratory study indicated that feeding another person using the robotic arm was an enjoyable social experience, and that it could potentially serve as an ice-breaker between strangers. In a similar vein, Mitchell et al. (2015) designed an actuated dining table where two people can eat together. The table lowers the plate of the person who is eating too fast, and raises the plate of the other so that diners' eating speeds become aligned. According to the authors, misalignment in eating pace between co-diners can create social friction and discomfort, something their system aims to address. The concept of the interactive table is also explored by Kado et al. (2010), who present a more abstract approach of agency with their *sociable dining table* (SDT). Users interact with the SDT by knocking on it which, according to the authors, serves as a minimal social cue to interact with “creatures”; actuated tableware such as a pot and a dish that can move around the table. An exploratory study indicated that users were able to guide the creatures around the table through the knocking interaction. While the social dimension of the installation deserves further study it is interesting to consider agency in a broader scope through interactions with robotic table wear. Li et al. (2018) explore ingestible sensors, i.e., microsystems that perform sensing inside the body. As an example they propose HeatCraft–two user interactive system which measures the internal body temperature of the one player and communicates it to the other player though thermal stimuli.

Humans often interact not only when eating but also when preparing food. Foodie by Wei and Nakatsu (2012) is an example of system that allows for joint design and creation of real food. The system is composed of the Food Creation Interface—a mobile app to design the food (e.g., to define its shape, color), and the Food Crafting Mechanism—a robot which crafts the designed food. In the use case scenario, multiple persons, by using their mobile devices, design new food together and send the project to another user whose robot generates edible food (e.g., a sandwich).

### 6.2. Tele-Dining

As demonstrated in Ferdous (2015), technology can enhance commensal experiences. During family mealtimes technology can scaffold and shape social interaction (Ferdous et al., 2016a). In Ferdous et al. (2016b), they present *TableTalk*, affording diners the possibility to bring together their photos, videos, audio, and other digital media to create a shared commensal technological experience. Nevertheless, the positive role of technology in these instances is focused on the traditional setting of the family dinner. Nawahdah and Inoue (2013) highlight that family dining is becoming very difficult today. Young people and the elderly are increasingly living independently, and working people tend to either travel frequently or work remotely, as also observed by Sellaeg and Chapman (2008). Hence, technology to enhance commensality can no longer only focus on the shared family dining table, but has to take into account distal interactions. Commensality can be re-introduced by exploring the possibility of what is called *remote commensality* (Foley-Fisher et al., 2010; Wei et al., 2011a; Grevet et al., 2012; Komaromi Haque, 2016). An example of a system aimed at creating a sense of remote commensality is the *KIZUNA* system (Nawahdah and Inoue, 2013; Inoue and Nawahdah, 2014). The system enables asynchronous dining interaction between people living in different time zones. The idea is that a person can experience remote co-dining with another person by watching a pre-recorded video of the other person's dining. The system works by separately recording the dining actions of two persons dining at different times and plays back these recordings by modulating the playback time to ensure the synchronization between the real and the recorded person. As the authors highlight in their work, it is not enough that people can merely watch another person dining, as it also happens, for example, with the *Cu-later* (Tsujita et al., 2010), to have the illusion of co-dining. It is the synchronization between their actions that contributes to achieving this illusion. The *KIZUNA* system was validated through questionnaires asking participants to rate the sense of presence they perceived from the remote (pre-recorded) person and the overall satisfaction of communication. The test had two conditions, one in which participants had dinner while watching a pre-recorded video of someone else eating and another one using a Wizard-of-Oz approach to simulate the *KIZUNA system*. Results showed that the system was preferred both in terms of presence perception and overall satisfaction of communication.

Similarly, Heidrich et al. (2012) presented the *Room XT* concept which consists of a wall-sized projection, head-tracking, and 3D rendering to create the illusion of sitting across from another person at a table. In this setup, head-tracking would allow for the projection to be adjusted to the point-of-view of the person looking at the projection to create the illusions of depth. The concept was implemented in a scaled-down setup using a computer monitor and Kinect sensor to demonstrate the potential of the depth illusion in a shared dining experience.

The importance of synchronized multimodal signals during remote co-dining is underlined by the work of Wei et al. (2011b) in the design of the *CoDine* system. The system consists of a large video screen, Kinect sensor, augmented table and tablecloth, and food printer. Remotely located diners can see each other through the screen and use the combination of the screen and Kinect sensor to engage in gesture-based interactions with the system through icons displayed on the screen. The icons can be selected to share messages through the tablecloth which, through thermochromic ink and Peltier elements, can change color to display simple shapes. Similarly, on-screen icons can be selected to create printed food shapes on the remotely located other's food items using the food printer. Finally, the augmented table is embedded with a movable magnet that allows a diner to remotely move another person's tableware, the idea being that this enables a form of sharing that is typically only possible during co-located dining. Aspects of the *CoDine* system were later implemented in *Foodie*, aimed at social interactions through printable food. Foodie by Wei and Cheok (2012) is an integrated system that allows for joint design and creation of real food (see section 6.1).

Where *CoDine* and *Foodie* enable interactions between two remotely located diners, the telematic dinner party system by Barden et al. (2012) allows remotely located groups of people to engage in interactions during dinner. After observational studies of dinner parties and an initial prototype design, the final design consisted of a set of round tables where three diners would gather around. Webcams were used to capture diners at one table and a projector was used to project visuals of remotely located diners. A projection area showed a visualization of a remotely located diner from the other table. In the center of each table a rotating platform was used to present food. Diners could physically rotate the platform on their table, which would result in an identical rotation in the platform of the remotely located table. The setup was evaluated during several dining scenarios (e.g., murder mystery dinner party), showing that communication was not as fluent as during a co-located dinner party. Conversely, participants did engage in playful behavior during the scenarios predominantly by manipulating the rotating platform, for example, while a remotely located diner was just about to reach for an item of food. The authors suggest that the element of playfulness helped overcome technical limitations while at the same time resulting in more of a performance than an actual dinner party.

### 6.3. Future Developments for Enhance Computational Commensality Between Humans

To conclude this section it is worth noticing that the systems mentioned in section 6.1 allow for some interaction between multiple humans. It can be as simple as trying to perform a better score in a game than all other competitors, or very complex scenarios requiring cooperation and which revolve around the topics of eating and food. The latter also show that the technology can change *eating* into a play and create an experience, which is enjoyable not because of the (consumed) food, but mainly due to connecting people by tasks that require joint actions in the physical space (Altarriba Bertran et al., 2018; Chisik et al., 2018). In this sense, for istance, works by Mehta et al. (2018) or Mitchell et al. (2015) are examples of CC, which would not be possible without using the technology (i.e., in traditional human-human setting).

The technology to enable remote commensality is becoming increasingly more sophisticated, and researchers have made headway in creating systems that allow individuals some form of visual communication and in some cases shared interaction with actual food items. However, it remains to be investigated whether or not these systems provide the same benefits of actual commensality—for example, the ones mentioned in section 2. In addition, these systems do not necessarily provide solutions for individuals experiencing (chronic) loneliness due to a lack of sufficiently satisfying social connections. Nevertheless, one may wonder whether the use of these systems could also be sought in shared dining with strangers, which could be seen as a potential approach to create new, hopefully in the end, satisfying, social relationships. The technology implemented in tele-dining systems could then also be used as conversation support technology to stimulate strangers to engage with each other socially (Otsuka and Inoue, 2012).

## 7. Final Discussion

Eating is a highly social activity, and so are everyday eating-related actions. For this reason, we believe computational models and techniques aimed at reading and understanding human non-verbal social interaction should pay attention to eating-related behaviors. In this survey, we hope to have provided an overview of existing psychological studies, approaches and technologies aimed at addressing, creating or augmenting commensality. The body of literature discussed shows that CC draws on many different fields. It is a complex, multi-disciplinary field of research still in its early stages. Therefore, a number of hiatuses remain that deserve to be addressed in future research.

Like current smart phone use, adding technology to the dining table will change social situations and rituals around the food consumed. Therefore, CC should take into account the impact of the technology once it is introduced into the dining sphere. For example, consider using a VR headset to visually augment food experiences. In such a situation, it becomes very difficult to share food experiences between co-diners due to the fact that the headset will make regular face-to-face interactions very difficult. Similarly, gamification, and augmentation technology in general, can also serve as potential distractors from food and food consumption. As an educational approach, one could question whether creating distractions is beneficial to long-term food enjoyment and healthy eating habits of children, for example. More generally, the argument can be put forth that any kind of technology that distracts from the actual food or genuine interaction during dinner can have potentially detrimental effects on food enjoyment, healthy eating, and conducive social eating habits.

At the same time, potential opportunities for sharing food and flavor experiences across distances (e.g., while connected through the internet) can be enabled by the same technologies, potentially providing commensal experiences where none were possible before. Future work on CC should carefully consider how the addition of technology to commensality will impact already existing social eating practices.

One way to have technology more seamlessly integrated into current dining practices is to move it into the background, and to adapt it to different eating situations as needed. However, technology to track and recognize food items is not fully implemented yet in many of the systems that have been discussed in this review. Therefore the manipulations, for example those aimed at guiding diners' behavior using AR, are typically hand-built to match the food items presented to participants. Considerable effort should be put into automatic recognition of food items in order to create seamless, automatic augmentations in an interaction loop where the food is recognized and the digital augmentation is automatically generated based on the recognition (e.g., to match or contrast some of the qualities of the food). Only through such integrations can these systems have a real place at the dining table, especially as far as commensal dining is concerned (see also previous point).

Works discussed in this survey that provide augmented food experiences often do so in controlled lab settings. One can question how strongly lab-based manipulations affect (commensal) food experiences in a real-life dining settings. Some restaurants do experiment with CC (e.g., Sublimotion, Ultra Violet), but there is currently a lack of research showing effects of technological augmentations on food experiences in ecologically valid settings. In relation to ecologically valid research, it is important to stress cultural aspects of commensality. There are strong differences between various cultures in commensal eating (Kittler et al., 2011; Counihan and Van Esterik, 2012; Anderson, 2014). However, little research in CC, be it related to food recognition, changing flavor experiences, or providing support through artificial social agents, takes into account cultural differences in a structural way. Therefore, it can be recommended for research to move away from focusing on WEIRD (Henrich et al., 2010) samples, and include more culturally diverse samples. At the very least, research in CC should be mindful of the fact that results may be limited to specific socio-cultural settings, and be difficult to generalize beyond that setting. As an example, in this context, nearly all commensal technologies listed in this paper (e.g., Kado et al., 2010; Mitchell et al., 2015) assume that eating is organized around the table (whether real, augmented or virtual). At the same time, it is well-known that people in several cultures eat and interact when eating without using such furniture. Consequently, it might be important also to develop culture-specific CC.

From a more technological perspective, it is important to carefully consider the validity of computational models of human-human behaviors at the table. Existing models dedicated to social signal processing, for example, leadership (Beyan et al., 2018; Niewiadomski et al., 2018), cohesion (Hung and Gatica-Perez, 2010), and turn-taking (de Kok and Heylen, 2009) might not necessarily be appropriate for analyzing behaviors in commensal scenarios. Indeed, when eating together, humans perform at least two different activities in parallel: eating and socializing. Both of these activities could be considered to interfere with each other. For example, when chewing or focusing on the food on the plate social behaviors that are typical in non-commensal social settings can be disrupted (e.g., turn-taking). In addition, the non-verbal behaviors and the communication with eating partners are limited by the position at the table and the distance to the interlocutors. These unique aspects of social interactions that occur while consuming food highlight the need to build new, multimodal corpora of commensality. Here, it is important to take into account spontaneous behavioral aspects related to the food specifically (e.g., food recognition, mastication) as well as the social behaviors that occur between co-located humans.

Indeed, such models may be a requirement to create truly social interactions with artificial social entities such as assistive robots or virtual coaches. These social interactions should not only be focused on food, diet, and eating behaviors, to name but a few application areas covered in this review, but should include interactions around other topics, from small talk about the weather to discussing the day at work. Through such more complete social interactions the bond between the user and the artificial social entity can potentially be strengthened. Only when embodied computational systems, such as social robots, can participate on some level in all the complexities of social interactions during meal time can we move toward true CC.

To conclude, we have seen in this paper that even if technology is often integrated in eating practices already, there is still the need for technologies capable of reading and generating social signals that are associated with such practices. This should motivate HCI and AI researchers to give more attention to different social aspects of food related interactions. Our hope is that this work will contribute to kick off new research and strengthen existing research initiatives in diverse fields toward the creation of novel computational models dealing with commensal food preparation and consumption.

## Author Contributions

All authors contributed to the introduction and revised the manuscript. EC: took main charge of shaping food as a social phenomenon. GH: technology for augmented flavor experiences and final discussion. MM: technology for food and eating recognition and technology for fostering human-human interaction. RN: assistive technology and technology for fostering human-human interaction.

### Conflict of Interest

The authors declare that the research was conducted in the absence of any commercial or financial relationships that could be construed as a potential conflict of interest.

## Footnotes

^1^https://acm-fca.org/2018/07/01/future-of-computing-food-manifesto

^2^The term “commensality” was used previously in a narrower sense, among others, in works by Ferdous et al. (2016a) on the influence of existing technology (e.g., tablets, smart phones) in familial interaction, or by Grevet et al. (2012) on promoting social awareness around mealtimes through communication of the “eating”-related statuses to remote confederates. In this paper, we propose a broader perspective, which includes, for instance, social interaction not only with another human but also the technology itself (e.g., social robots).

^3^https://github.com/MarcBS/keras

^4^http://www.tangsoo.de

^5^https://www.sublimotionibiza.com

^6^https://uvbypp.cc
